# In Vivo Positive Magnetic Resonance Imaging Applications of Poly(methyl vinyl ether-alt-maleic acid)-coated Ultra-small Paramagnetic Gadolinium Oxide Nanoparticles

**DOI:** 10.3390/molecules25051159

**Published:** 2020-03-05

**Authors:** Mohammad Yaseen Ahmad, Md. Wasi Ahmad, Huan Yue, Son Long Ho, Ji Ae Park, Ki-Hye Jung, Hyunsil Cha, Shanti Marasini, Adibehalsadat Ghazanfari, Shuwen Liu, Tirusew Tegafaw, Kwon-Seok Chae, Yongmin Chang, Gang Ho Lee

**Affiliations:** 1 Department of Chemistry and Department of Nanoscience and Nanotechnology (DNN), College of Natural Sciences, Kyungpook National University (KNU), Taegu 41566, Korea; yaseen.knu@gmail.com (M.Y.A.); mwahmad@du.edu.om (M.W.A.); 20100819@hanmail.net (H.Y.); sonlongh@gmail.com (S.L.H.); shantimarasini.sm@gmail.com (S.M.); adibeh.ghazanfari@gmail.com (A.G.); liushuwen0701@gmail.com (S.L.); tegafawtirusew@yahoo.com (T.T.); 2 Division of RI-Convergence Research, Korea Institute of Radiological & Medical Science (KIRAMS), Seoul 01817, Korea; jpark@kirams.re.kr (J.A.P.); kihyessi@kirams.re.kr (K.-H.J.); 3 Department of Molecular Medicine and Medical & Biological Engineering and DNN, School of Medicine, KNU and Hospital, Taegu 41566, Korea; hscha1002@daum.net; 4 Department of Biology Education and DNN, Teachers’ College, KNU, Taegu 41566, Korea; kschae@knu.ac.kr

**Keywords:** poly (methyl vinyl ether-alt-maleic acid), ultra-small Gd_2_O_3_ nanoparticle, paramagnetic, T_1_ magnetic resonance imaging, contrast agent

## Abstract

The study of ultra-small paramagnetic gadolinium oxide (Gd_2_O_3_) nanoparticles (NPs) as in vivo positive (T_1_) magnetic resonance imaging (MRI) contrast agents is one of the most attractive fields in nanomedicine. The performance of the Gd_2_O_3_ NP imaging agents depends on the surface-coating materials. In this study, poly(methyl vinyl ether-alt-maleic acid) (PMVEMA) was used as a surface-coating polymer. The PMVEMA-coated paramagnetic ultra-small Gd_2_O_3_ NPs with an average particle diameter of 1.9 nm were synthesized using the one-pot polyol method. They exhibited excellent colloidal stability in water and good biocompatibility. They also showed a very high longitudinal water proton spin relaxivity (r_1_) value of 36.2 s^−1^mM^−1^ (r_2_/r_1_ = 2.0; r_2_ = transverse water proton spin relaxivity) under a 3.0 tesla MR field which is approximately 10 times higher than the r_1_ values of commercial molecular contrast agents. High positive contrast enhancements were observed in in vivo T_1_ MR images after intravenous administration of the NP solution sample, demonstrating its potential as a T_1_ MRI contrast agent.

## 1. Introduction

Nanoparticle (NP) imaging agents have attracted much attention because of their advanced imaging properties compared with those of conventional molecular agents [1,2,3,4,5,6]. In addition, NPs can deliver various functional materials such as drugs for advanced applications that exploit their large specific surface areas [7,8,9,10,11]. Therefore, NP imaging agents are considered core materials in nanomedicine where they can be used for both disease diagnosis and therapy [11,12,13,14]. 

Among NPs, ultra-small gadolinium oxide (Gd_2_O_3_) NPs are especially interesting because they are known to be potential high-performance positive (i.e., T_1_) magnetic resonance imaging (MRI) contrast agents because of their high longitudinal water proton spin relaxivity (r_1_) [15,16,17,18]. Their r_1_ values are much higher than those (i.e., 3.0–5.0 s^−1^mM^−1^) [19,20] of commercial molecular Gd-chelates. In particular, their r_1_ value is maximal at ultra-small particle diameters, ranging from 1.5 to 2.5 nm [21]. NPs in this size range are eligible for renal excretion and are, therefore, suitable for in vivo applications [22,23]. 

In nanomedicine, surface engineering of NPs is critical because the NPs should exhibit both good colloidal stability and good biocompatibility [24]. In this respect, surface-coating materials play an important role. A hydrophilic ligand is preferred for surface coating because it can impart high colloidal stability. Importantly, it can lead to a high r_1_ value because it enables numerous water molecules to access the Gd_2_O_3_ NPs [17,25]. Thus, diverse surface-coating materials should be explored to coat Gd_2_O_3_ NPs. In the present study, poly(methyl vinyl ether-alt-maleic acid) (PMVEMA) was used as a surface-coating material because it is a hydrophilic and biodegradable polymer [26,27,28]. It has been widely used in applications involving internal cellular drug delivery [26], oral drug delivery [27], and cell encapsulation [28]. PMVEMA has two carboxyl groups per monomer unit and consequently, numerous carboxyl groups per polymer. This feature implies that PMVEMA can strongly bind to the Gd_2_O_3_ NP surface through multiple coordination bonds between its numerous carboxyl groups as electron donors and numerous Gd^3+^ ions as electron acceptors on the NP surface. Therefore, excellent colloidal stability, biocompatibility, and relaxometric properties are expected. 

Here, a facile one-pot polyol synthesis was used to prepare the PMVEMA-coated ultra-small Gd_2_O_3_ NPs that were subsequently subject to various analyses, including measurements of their colloidal stability, in vitro cellular toxicity, and magnetic and relaxometric properties. Their effectiveness as a potential T_1_ MRI contrast agent was demonstrated by recording in vivo T_1_ MR images under a 3.0 tesla MR field. 

## 2. Results and Discussion

### 2.1. Particle Diameter, Hydrodynamic Diameter, and Crystal Structure 

**Figure 1:**
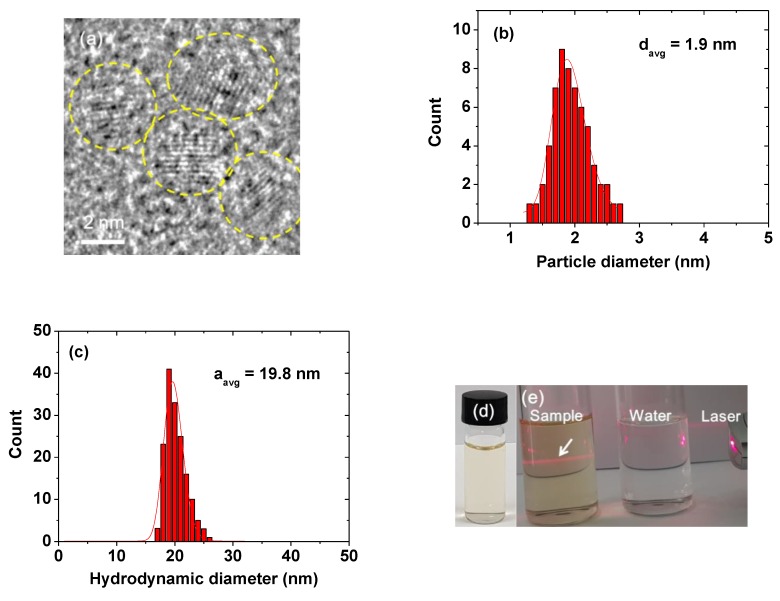
(**a**) HRTEM image (dotted circles indicate PMVEMA-coated ultra-small Gd_2_O_3_ NPs). (**b**) Log-normal function fit to the observed particle diameter distribution. (**c**) Log-normal function fit to the observed hydrodynamic diameter distribution. (**d**) Photograph of an aqueous solution sample, showing good colloidal stability. (**e**) Tyndall effect, indicating a colloidal dispersion: the left vial containing the solution sample showed laser light scattering by the PMVEMA-coated NP colloids (indicated with an arrow), whereas the right vial containing triple-distilled water did not.

The high-resolution transmission electron microscopy (HRTEM) image shows a nearly monodisperse particle size distribution (Figure 1a). The average particle diameter (d_avg_) was estimated to be 1.9 ± 0.1 nm from a log-normal function fit of the observed particle diameter distribution (Figure 1b and Table 1) (polydispersity index [29], PDI = 1.003, confirming monodispersity in particle diameter distribution). The average hydrodynamic diameter (a_avg_) was estimated to be 19.8 ± 0.1 nm from a log-normal function fit of the observed hydrodynamic diameter distribution (Figure 1c and Table 1) (PDI = 1.000, showing monodispersity in hydrodynamic diameter distribution). This large a_avg_ was due to the hydrophilic PMVEMA coating on the NP surface. Each PMVEMA (number-average molecular weight M_n_ ≈ 80 kDa) has 460 monomer units and each monomer has two COO^−^ groups (thus, a total of 920 COO^−^ groups per polymer). Consequently, the solution sample exhibited excellent colloidal stability. That is, the PMVEMA-coated NPs did not precipitate, as shown in the photograph of the solution sample in Figure 1d. The Tyndall effect shown in Figure 1e confirmed a colloidal dispersion of NPs where the left vial containing the PMVEMA-coated NPs in the figure exhibited laser light scattering because of the colloidal dispersion in solution, whereas the right vial containing triple-distilled water did not. 

**Figure 2:**
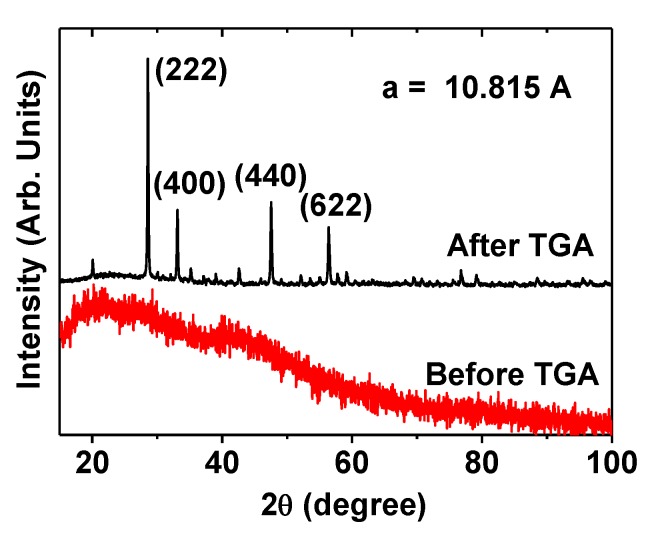
XRD patterns before (bottom spectrum) and after (top spectrum) TGA. All peaks in the XRD pattern after TGA were assigned with (hkl) Miller indices of cubic Gd_2_O_3_; only the intense peaks were representatively assigned. The estimated lattice constant, a = 10.815 Å.

The X-ray diffraction (XRD) pattern of the as-prepared powder sample was broad and amorphous (bottom XRD pattern in Figure 2) due to the ultra-small particle size of the Gd_2_O_3_ NPs [30]. However, after thermogravimetric analysis (TGA), the sample exhibited a cubic structure of bulk Gd_2_O_3_ (top XRD pattern in Figure 2), which was attributed to crystal growth and crystallization of the NPs during TGA to 900 °C, as previously reported [31]. The estimated lattice constant (a) of the TGA-treated powder sample was 10.815 Å, which is consistent with the reported value of 10.813 Å for Gd_2_O_3_ [32]. 

### 2.2. Surface-Coating with PMVEMA

**Figure 3:**
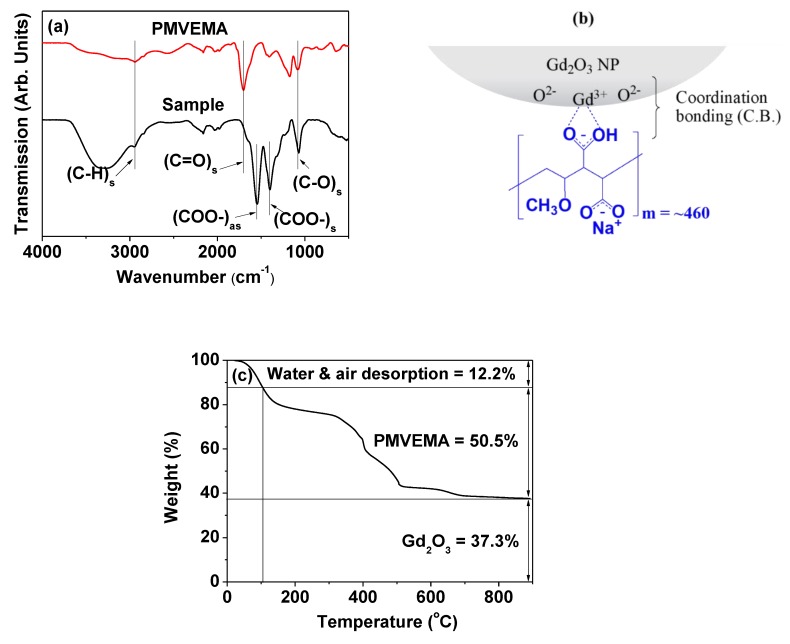
(**a**) FT-IR absorption spectra of the PMVEMA-coated ultra-small Gd_2_O_3_ NPs (bottom spectrum labeled as “Sample”) and free PMVEMA (top spectrum): the subscript “s” indicates a symmetric stretch and “as” indicates an antisymmetric stretch. (**b**) Surface-coating structure of PMVEMA on the Gd_2_O_3_ NP surface (one C.B. is presented at the figure, but numerous C.B.s exist between PMVEMA and the Gd_2_O_3_ NP). (**c**) TGA curve of the powder sample.

The surface coating of the ultra-small Gd_2_O_3_ NPs with PMVEMA was demonstrated by recording a Fourier transform infrared (FT-IR) absorption spectrum of the powder sample (bottom spectrum in Figure 3a). An FT-IR absorption spectrum of free PMVEMA was also recorded for reference (top spectrum in Figure 3a). As shown in Figure 3a, characteristic stretches of C–H at 2942 cm^−1^, COO^−^ at 1547 cm^−1^ (antisymmetric) and 1404 cm^−1^ (symmetric), and C–O at 1079 cm^−1^ were observed in the FT-IR absorption spectrum of the sample, confirming the successful surface coating of the Gd_2_O_3_ NPs with PMVEMA. Here, the COO^−^ stretching vibration of PMVEMA in the FT-IR absorption spectrum of the sample was split into symmetric and antisymmetric stretches of COO^−^ and was red-shifted with respect to the C=O stretching vibration at 1700 cm^−1^ in the spectrum of free PMVEMA. This red-shift was due to the bridge coordination bonding [33] of the COO^−^ to the Gd^3+^ of the NP (see Figure 3b for the surface-coating structure). Such bonding corresponds to hard base (COO^−^ group of PMVEMA)–hard acid (Gd^3+^ on the NP surface) bonding [34]. The observed large red-shifts indicate strong coordination bonds. Such red-shifts of the C=O stretch have been observed in other metallic oxides coated with ligands with –COOH groups, supporting our results [35]. Among the two COO^−^ groups per monomer unit of PMVEMA, the one opposite to the -OCH_3_ group likely participated in the coordination bonding due to its less steric hindrance from the -OCH_3_ group (Figure 3b). The other unconjugated COO^−^ is in the form of COO^−^Na^+^ because the PMVEMA-coated Gd_2_O_3_ NPs were synthesized at pH = ~10 and thus, showed COO^−^ peaks similar to those of the conjugated COO^−^ in the FT-IR absorption spectrum [36,37]. Multiple coordination bonds of PMVEMA to the NP are likely because each polymer contains numerous COO^−^ groups. Therefore, liberation of PMVEMA from the PMVEMA-coated NPs will not occur. This prediction is consistent with the observed excellent colloidal stability of the PMVEMA-coated NPs, which exhibited no precipitation. 

The amount (P) of surface-coated PMVEMA in wt % was estimated to be 50.5% from the TGA curve of PMVEMA-coated NPs after considering water and air desorption (12.2%) between room temperature and ~105 °C (Figure 3c and Table 1). The remaining mass was attributable to the Gd_2_O_3_ NPs (37.3%). The grafting density (σ), which corresponds to the average number of PMVEMA polymers coating a unit NP surface area [38], was estimated to be 0.05 nm^−2^ (Table 1) using the bulk density of Gd_2_O_3_ (7.41 g cm^−3^) [39], the wt % of PMVEMA estimated by TGA, and the d_avg_ estimated from HRTEM imaging. A high molecular weight of a polymer generally provides a low grafting density because large polymers occupy very large surface areas, as is the case in the present study. By multiplying the σ value by the NP surface area (πd_avg_^2^), the average number (N) of PMVEMA polymers coating a NP was estimated to be 0.57 (Table 1). This result indicates that one or two (mostly one) ultra-small Gd_2_O_3_ NPs were grafted with one PMVEMA through multiple coordination bonds, as previously described. 

### 2.3. In vitro Cellular Cytotoxicity Results

**Figure 4:**
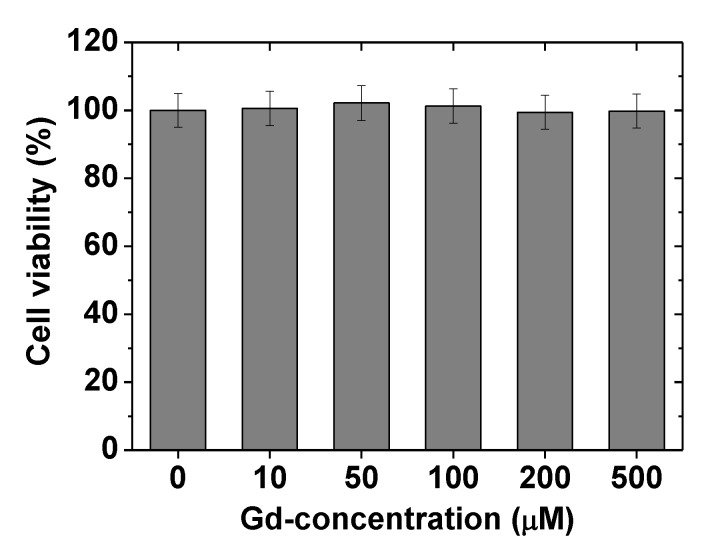
In vitro cellular cytotoxicity results of the PMVEMA-coated ultra-small Gd_2_O_3_ NPs in DU145 cells, showing negligible cellular toxicities at Gd-concentrations as high as 500 μM.

Free Gd^3+^ ions are toxic [40] and are well-known to cause nephrogenic systemic fibrosis [41]. Therefore, Gd_2_O_3_ NPs were coated with biocompatible PMVEMA. As shown in Figure 4, the PMVEMA-coated ultra-small Gd_2_O_3_ NPs exhibited very low toxicities at Gd-concentrations as high as 500 μM in DU145 cells from CellTiter-Glo Luminescent Cell Viability Assay [42], showing good biocompatibility. 

### 2.4. Magnetic Properties

**Figure 5:**
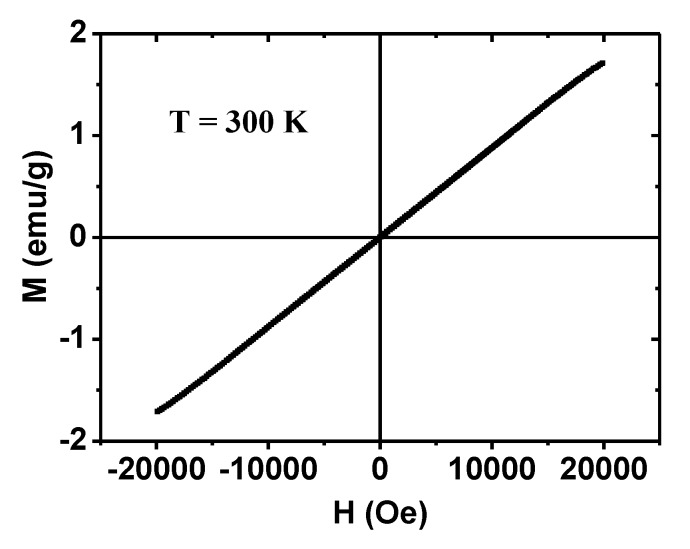
M–H curve of the PMVEMA-coated ultra-small Gd_2_O_3_ NPs at T = 300 K, showing paramagnetism. The M value is the net M value of the Gd_2_O_3_ NPs only without PMVEMA, which was estimated using their net mass obtained from the TGA curve.

Magnetic properties of the powder sample were characterized by recording a magnetization (M) versus applied (H) (i.e., M–H) curve (−2.0 tesla ≤ H ≤ 2.0 tesla) at temperature (T) = 300 K (Figure 5). The PMVEMA-coated ultra-small Gd_2_O_3_ NPs were paramagnetic (i.e., no hysteresis, zero coercivity, low M value, and zero remanence in the M−H curve), similar to the bulk material [43,44]. If the NPs were as superparamagnetic as iron oxide NPs [11,14], a high saturation magnetization should have been observed in the M-H curve. Furthermore, no phase transition had been previously observed in the M-T curve [31], due to paramagnetism of the NPs. The measured M value was mass-corrected using the net mass of the Gd_2_O_3_ NPs without PMVEMA, as estimated by TGA. From the mass-corrected M−H curve, the net M value of the Gd_2_O_3_ NPs at 2.0 tesla was estimated to be 1.71 emug^−^^1^ (Table 1). This appreciable value is due to the high electron-spin magnetic moment (S = 7/2) of Gd^3+^, which has seven unpaired 4f-electrons. 

### 2.5. Water Proton Spin Relaxivities

**Figure 6:**
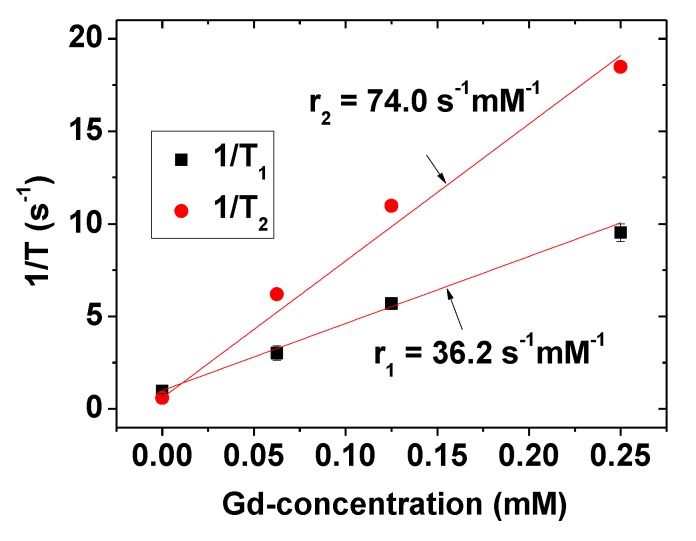
Plots of 1/T_1_ and 1/T_2_ as a function of the Gd-concentration. The slopes correspond to r_1_ and r_2_ values, respectively.

The longitudinal (T_1_) and transverse (T_2_) water proton spin relaxation times were acquired for various Gd-concentrations under a 3.0 tesla MR field. The longitudinal (r_1_) and transverse (r_2_) water proton spin relaxivities were then estimated to be 36.2 ± 1.4 and 74.0 ± 0.7 s^−1^mM^−1^ (r_2_/r_1_ = 2.0) from the slopes of 1/T_1_ and 1/T_2_ curves plotted as a function of the Gd-concentration, respectively (Figure 6 and Table 1). The confidence interval for r_1_ was estimated to be between 34.2 and 38.1 s^−1^mM^−1^ and that for r_2_ was estimated to be between 73.0 and 75.0 s^−1^mM^−1^ at a 95% confidence level. These results indicate that the PMVEMA-coated ultra-small Gd_2_O_3_ NPs strongly induce both T_1_ and T_2_ water proton spin relaxations. Notably, the observed r_1_ value is 10 times greater than the values [19,20] reported for commercial molecular Gd-chelates. The results imply that the PMVEMA-coated ultra-small Gd_2_O_3_ NPs will be a powerful T_1_ MRI contrast agent. 

The aforementioned excellent relaxometric properties are largely attributable to the hydrophilic PMVEMA coating because it can attract numerous water molecules and bind them close to the ultra-small Gd_2_O_3_ NPs. Under this condition, the large electron-spin magnetic moment (S = 7/2) of Gd^3+^ can strongly induce T_1_ water proton spin relaxation (inner-sphere mechanism) and the appreciable magnetization value of the Gd_2_O_3_ NPs can strongly induce T_2_ water proton spin relaxation (outer-sphere mechanism) [20,45], resulting in high r_1_ and r_2_ values, respectively. 

### 2.6. In vivo Contrast Enhancements in T_1_ MR Images 

**Figure 7:**
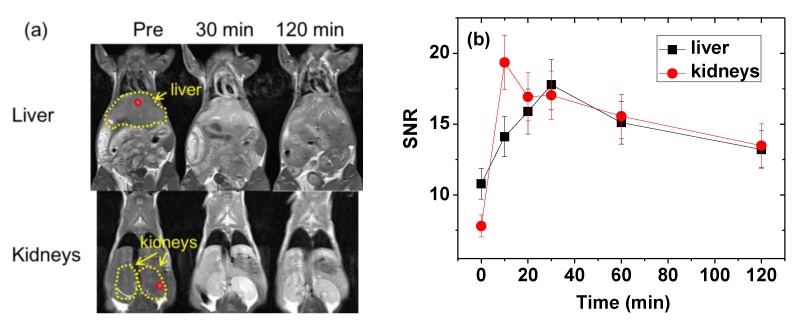
(**a**) In vivo T_1_ MR images of a mouse under a 3.0 tesla MR field before (labeled as “Pre”) and after intravenous administration of the solution sample into the mouse’s tail: the small circles indicate ROIs and the dotted circles indicate the liver and kidneys. The administration dose was ~0.1 mmol Gd/kg. (**b**) SNR plots of the ROIs in the liver and kidneys of the mouse as a function of time (SNR: signal-to-noise ratio; ROI: region-of-interest).

The effectiveness of the PMVEMA-coated ultra-small Gd_2_O_3_ NPs as a T_1_ MRI contrast agent was demonstrated by taking in vivo T_1_ MR images in a mouse under a 3.0 tesla MR field. As shown in Figure 7a, positive (i.e., brighter) contrast enhancements were observed in the liver and kidneys after intravenous administration of the aqueous solution sample into the mouse’s tail. To clearly show the contrast changes with time, signal-to-noise ratios (SNRs) of regions-of-interest (ROIs) in the liver and kidneys were plotted as a function of time, revealing that the contrasts initially increased, reached maxima, and then decreased with time in both organs (Figure 7b). These contrast changes are similar to those of molecular contrast agents, which generally excrete through the renal system within a few hours after intravenous administration. This molecular behavior is likely due to the ultra-small particle size of the NPs [22,23]. These results indicate that the PMVEMA-coated ultra-small Gd_2_O_3_ NPs are a potential T_1_ MRI contrast agent. 

## 3. Materials and Methods 

PMVEMA (M_n_ ≈ 80 kDa), GdCl_3_·*x*H_2_O (99.9%), NaOH (> 99.9%), and triethylene glycol (TEG) (99%) were purchased from Sigma-Aldrich (St. Louis, MI, USA) and used as received. Ethanol (>99%) was purchased from Duksan Chemical, South Korea and was used as received for the initial washing of the synthesized NPs. Triple-distilled water was used for the final washing of the synthesized NPs. 

**Figure 8:**
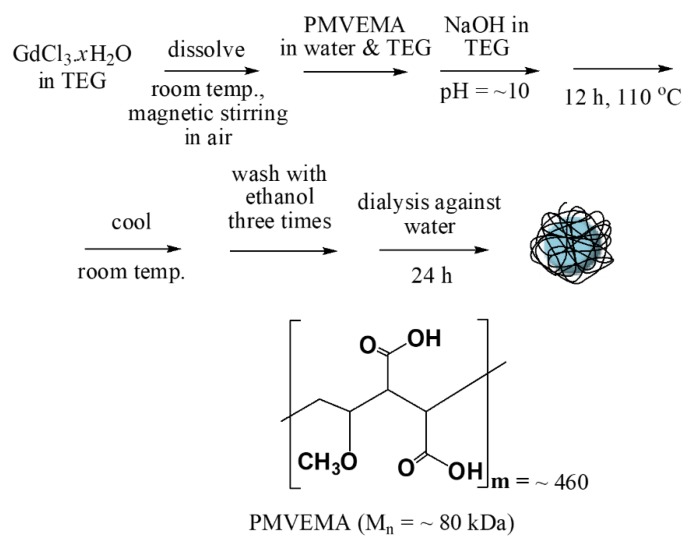
Reaction scheme for the one-pot polyol synthesis of PMVEMA-coated ultra-small Gd_2_O_3_ NPs and the molecular structure of PMVEMA.

A general scheme for the one-pot polyol synthesis of the PMVEMA-coated ultra-small Gd_2_O_3_ NPs is shown in Figure 8. Three separate solutions were prepared: (1) a precursor solution composed of 2 mmol of GdCl_3_·*x*H_2_O dissolved in 20 mL of TEG under magnetic stirring to form a clear solution in a three-necked round bottom flask under atmospheric conditions, (2) a PMVEMA solution containing 0.01 mmol of PMVEMA dissolved in 10 mL of triple-distilled water and 20 mL of TEG, and (3) a NaOH solution composed of 20 mmol of NaOH in 20 mL of TEG. Solution (2) was slowly added to solution (1) and the resulting mixture solution was magnetically stirred for 30 min. The solution (3) was then slowly added to the aforementioned mixture solution until the pH of the solution reached ~10. The reaction solution was then magnetically stirred at 110 °C for 12 h before cooling to room temperature. The product solution was washed with ethanol three times to remove unreacted precursors, NaOH, PMVEMA, and TEG. To this end, 400 mL of ethanol was added to the product solution, which was then magnetically stirred for 10 min and stored in a refrigerator until the product NPs settled to the bottom of the beaker. The supernatant transparent solution was decanted and the remaining product solution was washed with ethanol, again using the same process. To remove ethanol from the product NPs, the product solution was dialyzed against 1 L of triple-distilled water using a dialysis tube (molecular weight cutoff ≈ 2000 Da) for 24 h with magnetic stirring. Meanwhile, the water was replaced with fresh triple-distilled water every 8 h. One-half of the sample was dried in air to obtain a powder and the other half of the sample was diluted in triple-distilled water to prepare an aqueous NP suspension sample (>20 mM Gd). 

The particle diameter of the synthesized NPs was measured using an HRTEM instrument (Titan G2 ChemiSTEM CS Probe, FEI, Hillsboro, OR, USA) operated at 200 kV. Samples for HRTEM observation were prepared by dropping the solution sample diluted in ethanol onto a carbon film supported on a 200-mesh copper grid (PELCO No.160, TED PELLA, Inc., Redding, CA, USA) placed on filter paper. A dynamic light scattering particle size analyzer (UPA150, Microtrac) was used to measure the hydrodynamic diameter of the synthesized NPs. A dilute solution sample in triple-distilled water (<0.01 mM Gd) was used for the hydrodynamic diameter measurement. The crystal structure of the powder sample before and after TGA was measured using a powder XRD spectrometer (X-PERT PRO MRD, Philips) equipped with a CuKα (λ = 1.54184 Å) radiation source. The scanning step and scan range in 2θ were 0.033° and 15–100°, respectively. The attachment of PMVEMA to the Gd_2_O_3_ NP surfaces was investigated by recording FT-IR absorption spectra (Galaxy 7020A, Mattson Instruments, Inc., Madison, WI, USA) in the range of 400–4000 cm^−1^. The powder sample was dried on a hot plate at ~40 °C for one week to remove moisture and was then pelletized in KBr. The surface-coating amount was estimated by recording a TGA curve (SDT-Q 600, TA Instruments, New Castle, DE, USA). Because organic compounds burn out below 400 °C, the TGA curve of the powder sample was recorded in the temperature range from room temperature to 900 °C under flowing air. The amount of surface coating was estimated from the mass loss indicated in the TGA curve after subtraction of the initial mass drop between room temperature and ~105 °C as a result of water and air desorption. The Gd-concentration of the aqueous solution sample was determined using an inductively coupled plasma atomic emission spectrometer (IRIS/AP, Thermo Jarrell Ash Co., Waltham, MA, USA). A vibrating sample magnetometer (7407-S, Lake Shore Cryotronics, Inc., Westerville, OH, USA) was used to characterize the magnetic properties of the powder sample by recording its M−H curve (−2.0 tesla ≤ H ≤ 2.0 tesla) at 300 K. For measurements, 20–30 mg of the powder sample was used. The net M value of the sample (i.e., only Gd_2_O_3_ NPs without PMVEMA) was estimated using the net mass of the Gd_2_O_3_ NPs extracted from the TGA curve. 

The T_1_ and T_2_ water proton spin relaxation times were measured using a 3.0 tesla MRI scanner (MAGNETOM Trio Tim, Siemens, Munchen, Bayern, Germany). Four aqueous dilute solutions (0.25, 0.125, 0.0625, and 0.0 mM Gd) were prepared by the dilution of the concentrated solution sample with triple-distilled water. These diluted solutions were then used to measure both the T_1_ and T_2_ relaxation times. The T_1_ relaxation time measurements were conducted using an inversion recovery method. In this method, the inversion time was varied at 3.0 tesla and the MR images were acquired at 35 different inversion times in the range from 50 to 1750 ms. The T_1_ relaxation times were then obtained from the non-linear least-square fits to the measured signal intensities at various inversion times. For the measurements of T_2_ relaxation times, the Carr-Purcell-Meiboom-Gill pulse sequence was used for multiple spin-echo measurements. Thirty-four images were acquired at 34 different echo times in the range from 10 to 1900 ms. The T_2_ relaxation times were obtained from nonlinear least-square fits of the mean pixel values for the multiple spin-echo measurements at various echo times. The r_1_ and r_2_ water proton spin relaxivities were then estimated from the slopes of plots of 1/T_1_ and 1/T_2_, respectively, versus the Gd-concentration. 

The in vitro cytotoxicity of the synthesized NPs was measured using a CellTiter-Glo Luminescent Cell Viability Assay (Promega, Madison, WI, USA). To this end, the intracellular adenosine triphosphate was quantified using a luminometer (Victor 3, Perkin Elmer). The human prostate cancer (DU145) cell line was used as test cells. Cells were seeded onto a separate 24-well cell culture plate and incubated for 24 h (5 × 10^4^ cell density, 500 μL cells/well, 5% CO_2_, and 37 °C). Five dilute solution samples (i.e., 10, 50, 100, 200, and 500 μM Gd) were prepared by the dilution of the concentrated solution sample with a sterile phosphate-buffered saline solution. Each of the test cells was then treated with ~2 μL of each diluted solution sample. The treated cells were incubated for 48 h. Cell viability measurements were repeated twice to obtain average cell viabilities, which were then normalized with respect to that of untreated control cells (i.e., 0.0 M Gd). 

In vivo MRI studies using mice were performed in accordance with the rules and regulations of the animal research committee of the Korea Institute of Radiological and Medical Science. In vivo T_1_ MR images were acquired using the same MRI scanner used for the relaxometric measurements. For imaging, the mice (Balb/c nude male) (20–30 g) were anesthetized using 1.5% isoflurane in oxygen. Measurements were made before and after administration of the solution sample into the mouse’s tail veins. The administration dose was typically ~0.1 mmol Gd/kg. After measurement, the mice were revived from anesthesia and placed in a cage with free access to food and water. During measurements, the temperature of the mice was maintained at ~37 °C using a warm water blanket. The parameters used for the measurements were as follows: H = 3.0 tesla; temperature = 37 °C; number of acquisitions = 3; field of view = 60 mm; phase field of view = 1; matrix size = 256 × 256; slice thickness = 1 mm; spacing gap = 1.1 mm; number of slices = 24; pixel bandwidth = 15.63 Hz; repetition time = 564 ms; and echo time = 12 ms. 

## 4. Conclusions

In this study, a facile one-pot polyol synthesis of PMVEMA-coated ultra-small paramagnetic Gd_2_O_3_ NPs was presented. The synthesized NPs were applied to T_1_ MRI as a contrast agent. The results are summarized as follows. 

(1) The synthesized NPs exhibited a nearly monodisperse particle diameter distribution, with a d_avg_ of 1.9 nm and an a_avg_ of 19.8 nm. They were paramagnetic, with a net magnetization value (Gd_2_O_3_ NPs only, without PMVEMA) of 1.71 emu/g at 2.0 tesla and 300 K. 

(2) PMVEMA was strongly bonded to the ultra-small Gd_2_O_3_ NP surface through multiple coordination bonds between its numerous carboxyl groups and numerous Gd^3+^ on the NP surface. These strong multiple bonds and abundant carboxyl groups of the PMVEMA imparted the NPs with good colloidal stability (i.e., no precipitation), good biocompatibility (i.e., negligible cellular toxicities), and excellent relaxometric properties (i.e., r_1_ = 36.2 s^−1^ mM^−1^, and r_2_ = 74.0 s^−1^ mM^−1^) in which the r_1_ value was approximately 10 times greater than those of commercial molecular contrast agents. 

(3) The PMVEMA-coated ultra-small Gd_2_O_3_ NPs exhibited high positive contrasts in in vivo T_1_ MR images after intravenous administration, demonstrating their effectiveness as a T_1_ MRI contrast agent. 

## Figures and Tables

**Table 1:** Summary of the properties of PMVEMA-coated ultra-small Gd_2_O_3_ NPs.

d_avg_ (nm)	a_avg_ (nm)	Surface-Coating Amount			Magnetic Properties		Water Proton Spin Relaxivities under a 3.0 Tesla MR Field	
		P (wt %)	σ (nm^−2^)	N	Magnetism	M at 2.0 Tesla and 300K (emu/g)	r_1_ (s^−1^mM^−1^)	r_2_ (s^−1^mM^−1^)
1.9 ± 0.1	19.8 ± 0.1	50.5	0.05	0.57	Paramagnetic	1.71	36.2 ± 1.4	74.0 ± 0.7
